# Detecting spread of SARS‐CoV‐2 variants using PowerChek SARS‐CoV‐2 S‐gene mutation detection kit

**DOI:** 10.1002/jcla.24567

**Published:** 2022-06-24

**Authors:** Hyun Jin Kim, Seon Young Kim, Gye Cheol Kwon, Qute Choi

**Affiliations:** ^1^ Department of Laboratory Medicine Chungnam National University School of Medicine Daejeon South Korea; ^2^ Department of Laboratory Medicine Chungnam National University Sejong Hospital Sejong South Korea; ^3^ Department of Laboratory Medicine Chungnam National University Hospital Daejeon South Korea

Dear Editor,

Since detecting severe acute respiratory syndrome coronavirus 2 (SARS‐CoV‐2) in late December 2019, various genetic variants have been reported.^1^, ^2^, ^3^, ^4^, ^5^ Among them, the variants that were first reported in the United Kingdom (WHO label: Alpha lineage B.1.1.7),^6^, ^7^ South Africa (WHO label: Beta lineage B.1.351),^8^ Brazil (WHO label: Gamma lineage P.1),^9^ and India (WHO label: Delta lineage B.1.617.2)^10^ were of global public health significance.

Since SARS‐CoV‐2 variants easily spread, it is important to detect them quickly. However, rapid detection of SARS‐CoV‐2 variants is challenging. Variant detection through sequencing is time‐consuming and requires specific equipment. Some studies have detected variants using conventional reverse transcription‐polymerase chain reaction (RT‐PCR) following additional variant‐specific RT‐PCR with probes targeting variants in the spike glycoprotein.^11^, ^12^ Although these methods are faster than sequencing methods, they are performed in two steps. Therefore, a simpler test method is needed.

The recently developed PowerChek SARS‐CoV‐2 S‐gene mutation detection kit (Ver2.3) (Kogene Biotech, Seoul, Korea) is a rapid kit that detects SARS‐CoV‐2 variants using multiplex real‐time RT‐PCR.^13^ It can detect SARS‐CoV‐2 variants, including these variants from the United Kingdom (B. 1.1.7), South Africa (B. 1.351), Brazil/Japan (P. 1), and the United Kingdom (B. 1.525)/Brazil (P. 2). This study aimed to demonstrate the usefulness of the PowerChek SARS‐CoV‐2 S‐gene mutation detection kit in screening for SARS‐CoV‐2 variants.

From January to August 2021, 160 samples were confirmed to be SARS‐CoV‐2 positive using the PowerChek SARS‐CoV‐2 real‐time PCR Kit (Kogene Biotech, Seoul, Korea) in our routine laboratory tests. RNA extracted from these samples was screened for SARS‐CoV‐2 variants using the PowerChek SARS‐CoV‐2 S‐gene mutation detection kit, following the manufacturer's instructions. The kit consists of two sets of primer/probe mixes (R6908QP1 and R6908QP2), two sets of controls (R6908QC1 and R6908QC2), and an RT‐PCR master mix. Two sets of probes can detect six variants of the S gene: N501Y, K417N, E484K, P681R, E484Q, and L452R. By combining these six variants of the S gene, eight SARS‐CoV‐2 variants could be screened. Briefly, detecting only N501Y implied the United Kingdom (B.1.1.7) variant. Detecting N501Y, K417N, and E484K implied the South Africa (B.1.351) variant. Detecting N501Y and E484K implied the Brazil/Japan (P.1) variant. Detecting P681R and L452R implied the India (B.1.617.2) variant. The India (B.1.617), India (B.1.617.1/3), California (B.1.427/429), and New York (B.1.526.1) variants were determined by using other combinations according to the manufacturer's instructions. After screening, direct sequencing was performed for the final identification of each lineage. Sequencing was performed to identify the 69/70 deletion of the N‐terminal domain and the N501Y, L452R, E484K, K417N, and P681R of the receptor‐binding domain. The primers used are summarized in Table 1.

**TABLE 1:** The information of sequencing primers used in this study

Primer name	Forward sequence (5′‐3′)	Reverse sequence (5′‐3′)	Detection
Sequencing Primer_1	CACACGTGGTGTTTATTACCCT	GTTAGACTTCTCAGTGGAAGCA	69/70 deletion
Sequencing Primer_2	GAGGTGATGAAGTCAGACAAATCG	CTCTGTATGGTTGGTAACCAACA	E484K, E484Q, K417N, L452R, and N501Y
Sequencing Primer_3	CTCCTACTTGGCGTGTTTATTCT	CTACTGATGTCTTGGTCATAGACA	P681R

The results of real‐time PCR and sequencing are presented in Table 2. The most observed lineage was the B.1.617.2 (Delta) variant, with an incidence rate of 34% (54/160). All samples screened for B.1.617.2 by real‐time PCR were confirmed as B.1.617.2 on sequencing. The second most observed lineage was SARS‐CoV‐2 without mutation (29%), in which the screening and confirmation test results were totally concordant. Of the 40 samples screened for B.1.525 (Eta)/P.2 (Zeta) variants by real‐time PCR, 35 (22%) were identified as P.2 (Zeta) variants and 5 (3%) as B.1.525 (Eta) variants by sequencing. The incidence rate of the B.1.1.7 (Alpha) variant was 8% (13/160), and the two test results were consistent. Six samples (4%) were identified as B.1.427/1.429 (Epsilon)/B.1.526.1 (Iota) using real‐time PCR, and it was not possible to distinguish each variant by sequencing. All results of the 160 samples are summarized in the Table S1.

**TABLE 2:** The results of real‐time PCR and sanger sequencing observed in this study

Real‐time PCR						Sanger sequencing					Variants	No. of samples (%)
P1			P2			69/70 deletion	Receptor‐binding domain (RBD)					
N501Y	K417N	E484K	P681R	E484Q	L452R		N501Y	L452R	E484K	P681R		
−	−	−	−	−	−	−	−	−	−	−	SARS‐CoV‐2	47 (29%)
+	−	−	−	−	−	+	+^a^	−	−	−	B.1.1.7 (Alpha)	13 (8%)
−	−	−	+	−	+	−	−	+^‡^	−	+^¶^	B.1.617.2 (Delta)	54 (33%)
−	−	−	−	−	+	−	−	+^‡^	−	−	B.1.427/B.1.429 (Epsilon)/B.1.526.1 (Iota)	6 (4%)
−	−	+	−	−	−	+	−	−	+^§^	−	B.1.525 (Eta)	5 (3%)
−	−	+	−	−	−	−	−	−	+^§^	−	P.2 (Zeta)	35 (22%)

^a^ c.1501A > T.

^‡^ c.1355 T > G.

^§^ c.1450G > A.

^¶^ c.2042C > G.

In this study, we confirmed that the overall concordance rate between the PowerChek SARS‐CoV‐2 S‐gene mutation detection kit and sequencing was 100% (160/160). In 75% of the samples (120/160), both the PowerChek kit and sequencing results were exactly matched. Moreover, in 25% of these samples (40/160), sequencing results were more refined: the B.1.525 (Eta)/P.2 (Zeta) variants were distinguished by sequencing. Most variants detected in this study could be identified using real‐time PCR without sequencing.

In conclusion, we demonstrated the distribution of SARS‐CoV‐2 variants in our region using the PowerChek SARS‐CoV‐2 S‐gene mutation detection kit. Most variants could be detected in a single step without sequencing. The PowerChek SARS‐CoV‐2 S‐gene mutation detection kit can be easily used to screen SARS‐CoV‐2 variants.

## CONFLICT OF INTEREST

The authors of this letter have no relevant financial or other relationships to disclose.

## ETHICAL APPROVAL

This study was approved by the Institutional Review Board of Chungnam National University Sejong Hospital, Sejong, Korea (approval number: 2021–05‐021).

## Supporting information


Table S1

## Data Availability

The data used to support the findings of this study are included in the article.
