# Supplementary material for: Detecting spread of SARS‐CoV‐2 variants using PowerChek SARS‐CoV‐2 S‐gene mutation detection kit
Source: J Clin Lab Anal. 2022 Jun 24;36(8):e24567. doi: 10.1002/jcla.24567 (PMC9349760; doi:10.1002/jcla.24567)
Supplement: Supplementary file 1 — Table S1 [file JCLA-36-e24567-s001.docx]

Supplementary Table

| No. | Real-time PCR | | | | | | | | | Sanger sequencing | | | | | | | | |  |
| --- | --- | --- | --- | --- | --- | --- | --- | --- | --- | --- | --- | --- | --- | --- | --- | --- | --- | --- | --- |
|  | P1 | | | P2 | | | | Variants | | 69/70 deletion | | Receptor bi-ing domain (RBD) | | | | | Variants | |  |
|  | N501Y | K417N | E484K | | P681R | E484Q | L452R | |  | |  | | N501Y | L452R | E484K | P681R | |  | |
| 1 | - | - | - | | - | - | - | | SARS-CoV-2 | | - | | A | T | G | C | | SARS-CoV-2 | |
| 2 | - | - | - | | - | - | - | | SARS-CoV-2 | | - | | A | T | G | C | | SARS-CoV-2 | |
| 3 | - | - | - | | - | - | - | | SARS-CoV-2 | | - | | A | T | G | C | | SARS-CoV-2 | |
| 4 | - | - | - | | - | - | - | | SARS-CoV-2 | | - | | A | T | G | C | | SARS-CoV-2 | |
| 5 | - | - | - | | - | - | - | | SARS-CoV-2 | | - | | A | T | G | C | | SARS-CoV-2 | |
| 6 | - | - | - | | - | - | - | | SARS-CoV-2 | | - | | A | T | G | C | | SARS-CoV-2 | |
| 7 | - | - | - | | - | - | + | | B.1.427 / B.1.429 (Epsilon) / B.1.526.1 (Iota) | | - | | A | G | G | C | | B.1.427 / B.1.429 (Epsilon) / B.1.526.1 (Iota) | |
| 8 | - | - | - | | - | - | - | | SARS-CoV-2 | | - | | A | T | G | C | | SARS-CoV-2 | |
| 9 | - | - | - | | - | - | + | | B.1.427 / B.1.429 (Epsilon) / B.1.526.1 (Iota) | | - | | A | G | G | C | | B.1.427 / B.1.429 (Epsilon) / B.1.526.1 (Iota) | |
| 10 | - | - | - | | - | - | - | | SARS-CoV-2 | | - | | A | T | G | C | | SARS-CoV-2 | |
| 11 | - | - | - | | - | - | + | | B.1.427 / B.1.429 (Epsilon) / B.1.526.1 (Iota) | | - | | A | G | G | C | | B.1.427 / B.1.429 (Epsilon) / B.1.526.1 (Iota) | |
| 12 | - | - | - | | - | - | - | | SARS-CoV-2 | | - | | A | T | G | C | | SARS-CoV-2 | |
| 13 | - | - | - | | - | - | - | | SARS-CoV-2 | | - | | A | T | G | C | | SARS-CoV-2 | |
| 14 | - | - | - | | - | - | - | | SARS-CoV-2 | | - | | A | T | G | C | | SARS-CoV-2 | |
| 15 | - | - | - | | - | - | - | | SARS-CoV-2 | | - | | A | T | G | C | | SARS-CoV-2 | |
| 16 | - | - | - | | - | - | - | | SARS-CoV-2 | | - | | A | T | G | C | | SARS-CoV-2 | |
| 17 | - | - | - | | - | - | - | | SARS-CoV-2 | | - | | A | T | G | C | | SARS-CoV-2 | |
| 18 | - | - | - | | - | - | - | | SARS-CoV-2 | | - | | A | T | G | C | | SARS-CoV-2 | |
| 19 | - | - | - | | - | - | - | | SARS-CoV-2 | | - | | A | T | G | C | | SARS-CoV-2 | |
| 20 | + | - | - | | - | - | - | | B.1.1.7 (Alpha) | | + | | T | T | G | C | | B.1.1.7 (Alpha) | |
| 21 | - | - | - | | - | - | - | | SARS-CoV-2 | | - | | A | T | G | C | | SARS-CoV-2 | |
| 22 | - | - | - | | - | - | - | | SARS-CoV-2 | | - | | A | T | G | C | | SARS-CoV-2 | |
| 23 | - | - | - | | - | - | - | | SARS-CoV-2 | | - | | A | T | G | C | | SARS-CoV-2 | |
| 24 | - | - | - | | - | - | - | | SARS-CoV-2 | | - | | A | T | G | C | | SARS-CoV-2 | |
| 25 | - | - | - | | - | - | - | | SARS-CoV-2 | | - | | A | T | G | C | | SARS-CoV-2 | |
| 26 | - | - | - | | - | - | - | | SARS-CoV-2 | | - | | A | T | G | C | | SARS-CoV-2 | |
| 27 | - | - | - | | - | - | - | | SARS-CoV-2 | | - | | A | T | G | C | | SARS-CoV-2 | |
| 28 | - | - | - | | - | - | - | | SARS-CoV-2 | | - | | A | T | G | C | | SARS-CoV-2 | |
| 29 | - | - | - | | - | - | - | | SARS-CoV-2 | | - | | A | T | G | C | | SARS-CoV-2 | |
| 30 | - | - | - | | - | - | - | | SARS-CoV-2 | | - | | A | T | G | C | | SARS-CoV-2 | |
| 31 | - | - | - | | - | - | - | | SARS-CoV-2 | | - | | A | T | G | C | | SARS-CoV-2 | |
| 32 | - | - | - | | - | - | - | | SARS-CoV-2 | | - | | A | T | G | C | | SARS-CoV-2 | |
| 33 | - | - | - | | - | - | - | | SARS-CoV-2 | | - | | A | T | G | C | | SARS-CoV-2 | |
| 34 | - | - | - | | - | - | - | | SARS-CoV-2 | | - | | A | T | G | C | | SARS-CoV-2 | |
| 35 | - | - | - | | - | - | - | | SARS-CoV-2 | | - | | A | T | G | C | | SARS-CoV-2 | |
| 36 | - | - | - | | - | - | - | | SARS-CoV-2 | | - | | A | T | G | C | | SARS-CoV-2 | |
| 37 | - | - | + | | - | - | - | | B.1.525 (Eta) / P.2 (Zeta) | | - | | A | T | A | C | | P.2 (Zeta) | |
| 38 | - | - | - | | - | - | - | | SARS-CoV-2 | | - | | A | T | G | C | | SARS-CoV-2 | |
| 39 | + | - | - | | - | - | - | | B.1.1.7 (Alpha) | | + | | T | T | G | C | | B.1.1.7 (Alpha) | |
| 40 | - | - | - | | - | - | - | | SARS-CoV-2 | | - | | A | T | G | C | | SARS-CoV-2 | |
| 41 | - | - | - | | - | - | - | | SARS-CoV-2 | | - | | A | T | G | C | | SARS-CoV-2 | |
| 42 | - | - | - | | - | - | + | | B.1.427 / B.1.429 (Epsilon) / B.1.526.1 (Iota) | | - | | A | G | G | C | | B.1.427 / B.1.429 (Epsilon) / B.1.526.1 (Iota) | |
| 43 | - | - | - | | - | - | - | | SARS-CoV-2 | | - | | A | T | G | C | | SARS-CoV-2 | |
| 44 | - | - | + | | - | - | - | | B.1.525 (Eta) / P.2 (Zeta) | | - | | A | T | A | C | | P.2 (Zeta) | |
| 45 | - | - | + | | - | - | - | | B.1.525 (Eta) / P.2 (Zeta) | | - | | A | T | A | C | | P.2 (Zeta) | |
| 46 | - | - | - | | - | - | - | | SARS-CoV-2 | | - | | A | T | G | C | | SARS-CoV-2 | |
| 47 | - | - | + | | - | - | - | | B.1.525 (Eta) / P.2 (Zeta) | | - | | A | T | A | C | | P.2 (Zeta) | |
| 48 | - | - | + | | - | - | - | | B.1.525 (Eta) / P.2 (Zeta) | | - | | A | T | A | C | | P.2 (Zeta) | |
| 49 | - | - | + | | - | - | - | | B.1.525 (Eta) / P.2 (Zeta) | | - | | A | T | A | C | | P.2 (Zeta) | |
| 50 | - | - | + | | - | - | - | | B.1.525 (Eta) / P.2 (Zeta) | | - | | A | T | A | C | | P.2 (Zeta) | |
| 51 | - | - | - | | - | - | - | | SARS-CoV-2 | | - | | A | T | G | C | | SARS-CoV-2 | |
| 52 | - | - | + | | - | - | - | | B.1.525 (Eta) / P.2 (Zeta) | | - | | A | T | A | C | | P.2 (Zeta) | |
| 53 | - | - | - | | - | - | + | | B.1.427 / B.1.429 (Epsilon) / B.1.526.1 (Iota) | | - | | A | G | G | C | | B.1.427 / B.1.429 (Epsilon) / B.1.526.1 (Iota) | |
| 54 | - | - | - | | - | - | - | | SARS-CoV-2 | | - | | A | T | G | C | | SARS-CoV-2 | |
| 55 | - | - | - | | - | - | + | | B.1.427 / B.1.429 (Epsilon) / B.1.526.1 (Iota) | | - | | A | G | G | C | | B.1.427 / B.1.429 (Epsilon) / B.1.526.1 (Iota) | |
| 56 | - | - | - | | - | - | - | | SARS-CoV-2 | | - | | A | T | G | C | | SARS-CoV-2 | |
| 57 | - | - | + | | - | - | - | | B.1.525 (Eta) / P.2 (Zeta) | | - | | A | T | A | C | | P.2 (Zeta) | |
| 58 | - | - | - | | - | - | - | | SARS-CoV-2 | | - | | A | T | G | C | | SARS-CoV-2 | |
| 59 | - | - | - | | - | - | - | | SARS-CoV-2 | | - | | A | T | G | C | | SARS-CoV-2 | |
| 60 | - | - | + | | - | - | - | | B.1.525 (Eta) / P.2 (Zeta) | | - | | A | T | A | C | | P.2 (Zeta) | |
| 61 | - | - | - | | - | - | - | | SARS-CoV-2 | | - | | A | T | G | C | | SARS-CoV-2 | |
| 62 | - | - | - | | - | - | - | | SARS-CoV-2 | | - | | A | T | G | C | | SARS-CoV-2 | |
| 63 | - | - | - | | - | - | - | | SARS-CoV-2 | | - | | A | T | G | C | | SARS-CoV-2 | |
| 64 | - | - | - | | - | - | - | | SARS-CoV-2 | | - | | A | T | G | C | | SARS-CoV-2 | |
| 65 | - | - | - | | - | - | - | | SARS-CoV-2 | | - | | A | T | G | C | | SARS-CoV-2 | |
| 66 | - | - | + | | - | - | - | | B.1.525 (Eta) / P.2 (Zeta) | | + | | A | T | A | C | | B.1.525 (Eta) | |
| 67 | - | - | + | | - | - | - | | B.1.525 (Eta) / P.2 (Zeta) | | + | | A | T | A | A | | B.1.525 (Eta) | |
| 68 | - | - | + | | - | - | - | | B.1.525 (Eta) / P.2 (Zeta) | | + | | A | T | A | A | | B.1.525 (Eta) | |
| 69 | - | - | + | | - | - | - | | B.1.525 (Eta) / P.2 (Zeta) | | - | | A | T | A | C | | P.2 (Zeta) | |
| 70 | - | - | + | | - | - | - | | B.1.525 (Eta) / P.2 (Zeta) | | - | | A | T | A | C | | P.2 (Zeta) | |
| 71 | - | - | + | | - | - | - | | B.1.525 (Eta) / P.2 (Zeta) | | - | | A | T | A | C | | P.2 (Zeta) | |
| 72 | - | - | + | | - | - | - | | B.1.525 (Eta) / P.2 (Zeta) | | + | | A | T | A | A | | B.1.525 (Eta) | |
| 73 | - | - | + | | - | - | - | | B.1.525 (Eta) / P.2 (Zeta) | | - | | A | T | A | C | | P.2 (Zeta) | |
| 74 | - | - | + | | - | - | - | | B.1.525 (Eta) / P.2 (Zeta) | | - | | A | T | A | C | | P.2 (Zeta) | |
| 75 | - | - | + | | - | - | - | | B.1.525 (Eta) / P.2 (Zeta) | | - | | A | T | A | C | | P.2 (Zeta) | |
| 76 | - | - | + | | - | - | - | | B.1.525 (Eta) / P.2 (Zeta) | | - | | A | T | A | C | | P.2 (Zeta) | |
| 77 | - | - | + | | - | - | - | | B.1.525 (Eta) / P.2 (Zeta) | | - | | A | T | A | C | | P.2 (Zeta) | |
| 78 | - | - | + | | - | - | - | | B.1.525 (Eta) / P.2 (Zeta) | | - | | A | T | A | C | | P.2 (Zeta) | |
| 79 | - | - | + | | - | - | - | | B.1.525 (Eta) / P.2 (Zeta) | | - | | A | T | A | C | | P.2 (Zeta) | |
| 80 | - | - | + | | - | - | - | | B.1.525 (Eta) / P.2 (Zeta) | | - | | A | T | A | C | | P.2 (Zeta) | |
| 81 | + | - | - | | - | - | - | | B.1.1.7 (Alpha) | | + | | T | T | G | A | | B.1.1.7 (Alpha) | |
| 82 | + | - | - | | - | - | - | | B.1.1.7 (Alpha) | | + | | T | T | G | A | | B.1.1.7 (Alpha) | |
| 83 | - | - | + | | - | - | - | | B.1.525 (Eta) / P.2 (Zeta) | | - | | A | T | A | C | | P.2 (Zeta) | |
| 84 | - | - | - | | + | - | + | | B.1.617.2 (Delta) | | - | | A | G | G | G | | B.1.617.2 (Delta) | |
| 85 | + | - | - | | - | - | - | | B.1.1.7 (Alpha) | | + | | T | T | G | A | | B.1.1.7 (Alpha) | |
| 86 | - | - | + | | - | - | - | | B.1.525 (Eta) / P.2 (Zeta) | | - | | A | T | A | C | | P.2 (Zeta) | |
| 87 | - | - | + | | - | - | - | | B.1.525 (Eta) / P.2 (Zeta) | | - | | A | T | A | C | | P.2 (Zeta) | |
| 88 | - | - | - | | + | - | + | | B.1.617.2 (Delta) | | - | | A | G | G | G | | B.1.617.2 (Delta) | |
| 89 | + | - | - | | - | - | - | | B.1.1.7 (Alpha) | | + | | T | T | G | A | | B.1.1.7 (Alpha) | |
| 90 | - | - | + | | - | - | - | | B.1.525 (Eta) / P.2 (Zeta) | | - | | A | T | A | C | | P.2 (Zeta) | |
| 91 | + | - | - | | - | - | - | | B.1.1.7 (Alpha) | | + | | T | T | G | A | | B.1.1.7 (Alpha) | |
| 92 | + | - | - | | - | - | - | | B.1.1.7 (Alpha) | | - | | T | T | G | A | | B.1.1.7 (Alpha) | |
| 93 | - | - | - | | + | - | + | | B.1.617.2 (Delta) | | - | | A | G | G | G | | B.1.617.2 (Delta) | |
| 94 | - | - | + | | - | - | - | | B.1.525 (Eta) / P.2 (Zeta) | | - | | A | T | A | C | | P.2 (Zeta) | |
| 95 | - | - | + | | - | - | - | | B.1.525 (Eta) / P.2 (Zeta) | | - | | A | T | A | C | | P.2 (Zeta) | |
| 96 | + | - | - | | - | - | - | | B.1.1.7 (Alpha) | | + | | T | T | G | A | | B.1.1.7 (Alpha) | |
| 97 | - | - | + | | - | - | - | | B.1.525 (Eta) / P.2 (Zeta) | | - | | A | T | A | C | | P.2 (Zeta) | |
| 98 | - | - | - | | + | - | + | | B.1.617.2 (Delta) | | - | | A | G | G | G | | B.1.617.2 (Delta) | |
| 99 | - | - | + | | - | - | - | | B.1.525 (Eta) / P.2 (Zeta) | | - | | A | T | A | C | | P.2 (Zeta) | |
| 100 | - | - | - | | + | - | + | | B.1.617.2 (Delta) | | - | | A | G | G | G | | B.1.617.2 (Delta) | |
| 101 | - | - | - | | + | - | + | | B.1.617.2 (Delta) | | - | | A | G | G | G | | B.1.617.2 (Delta) | |
| 102 | + | - | - | | - | - | - | | B.1.1.7 (Alpha) | | - | | T | T | G | A | | B.1.1.7 (Alpha) | |
| 103 | - | - | + | | - | - | - | | B.1.525 (Eta) / P.2 (Zeta) | | - | | A | T | A | C | | P.2 (Zeta) | |
| 104 | - | - | - | | + | - | + | | B.1.617.2 (Delta) | | - | | A | G | G | G | | B.1.617.2 (Delta) | |
| 105 | - | - | - | | + | - | + | | B.1.617.2 (Delta) | | - | | A | G | G | G | | B.1.617.2 (Delta) | |
| 106 | + | - | - | | - | - | - | | B.1.1.7 (Alpha) | | - | | T | T | G | A | | B.1.1.7 (Alpha) | |
| 107 | - | - | - | | + | - | + | | B.1.617.2 (Delta) | | - | | A | G | G | G | | B.1.617.2 (Delta) | |
| 108 | - | - | - | | + | - | + | | B.1.617.2 (Delta) | | - | | A | G | G | G | | B.1.617.2 (Delta) | |
| 109 | - | - | + | | - | - | - | | B.1.525 (Eta) / P.2 (Zeta) | | - | | A | T | A | C | | P.2 (Zeta) | |
| 110 | - | - | + | | - | - | - | | B.1.525 (Eta) / P.2 (Zeta) | | - | | A | T | A | C | | P.2 (Zeta) | |
| 111 | - | - | - | | + | - | + | | B.1.617.2 (Delta) | | - | | A | G | G | G | | B.1.617.2 (Delta) | |
| 112 | - | - | + | | - | - | - | | B.1.525 (Eta) / P.2 (Zeta) | | + | | A | T | A | C | | B.1.525 (Eta) | |
| 113 | - | - | - | | + | - | + | | B.1.617.2 (Delta) | | - | | A | G | G | G | | B.1.617.2 (Delta) | |
| 114 | - | - | - | | + | - | + | | B.1.617.2 (Delta) | | - | | A | G | G | G | | B.1.617.2 (Delta) | |
| 115 | - | - | - | | + | - | + | | B.1.617.2 (Delta) | | - | | A | G | G | G | | B.1.617.2 (Delta) | |
| 116 | - | - | + | | - | - | - | | B.1.525 (Eta) / P.2 (Zeta) | | - | | A | T | A | A | | P.2 (Zeta) | |
| 117 | - | - | - | | + | - | + | | B.1.617.2 (Delta) | | - | | A | G | G | G | | B.1.617.2 (Delta) | |
| 118 | - | - | - | | + | - | + | | B.1.617.2 (Delta) | | - | | A | G | G | G | | B.1.617.2 (Delta) | |
| 119 | - | - | - | | + | - | + | | B.1.617.2 (Delta) | | - | | A | G | G | G | | B.1.617.2 (Delta) | |
| 120 | - | - | + | | - | - | - | | B.1.525 (Eta) / P.2 (Zeta) | | - | | A | T | A | C | | P.2 (Zeta) | |
| 121 | - | - | - | | + | - | + | | B.1.617.2 (Delta) | | - | | A | G | G | G | | B.1.617.2 (Delta) | |
| 122 | - | - | - | | + | - | + | | B.1.617.2 (Delta) | | - | | A | G | G | G | | B.1.617.2 (Delta) | |
| 123 | - | - | - | | + | - | + | | B.1.617.2 (Delta) | | - | | A | G | G | G | | B.1.617.2 (Delta) | |
| 124 | - | - | - | | + | - | + | | B.1.617.2 (Delta) | | - | | A | G | G | G | | B.1.617.2 (Delta) | |
| 125 | - | - | + | | - | - | - | | B.1.525 (Eta) / P.2 (Zeta) | | - | | A | T | A | C | | P.2 (Zeta) | |
| 126 | - | - | - | | + | - | + | | B.1.617.2 (Delta) | | - | | A | G | G | G | | B.1.617.2 (Delta) | |
| 127 | - | - | - | | + | - | + | | B.1.617.2 (Delta) | | - | | A | G | G | G | | B.1.617.2 (Delta) | |
| 128 | - | - | - | | + | - | + | | B.1.617.2 (Delta) | | - | | A | G | G | G | | B.1.617.2 (Delta) | |
| 129 | - | - | - | | + | - | + | | B.1.617.2 (Delta) | | - | | A | G | G | G | | B.1.617.2 (Delta) | |
| 130 | - | - | - | | + | - | + | | B.1.617.2 (Delta) | | - | | A | G | G | G | | B.1.617.2 (Delta) | |
| 131 | - | - | - | | + | - | + | | B.1.617.2 (Delta) | | - | | A | G | G | G | | B.1.617.2 (Delta) | |
| 132 | + | - | - | | - | - | - | | B.1.1.7 (Alpha) | | - | | T | T | G | A | | B.1.1.7 (Alpha) | |
| 133 | - | - | - | | + | - | + | | B.1.617.2 (Delta) | | - | | A | G | G | G | | B.1.617.2 (Delta) | |
| 134 | - | - | - | | + | - | + | | B.1.617.2 (Delta) | | - | | A | G | G | G | | B.1.617.2 (Delta) | |
| 135 | - | - | - | | + | - | + | | B.1.617.2 (Delta) | | - | | A | G | G | G | | B.1.617.2 (Delta) | |
| 136 | - | - | - | | + | - | + | | B.1.617.2 (Delta) | | - | | A | G | G | G | | B.1.617.2 (Delta) | |
| 137 | - | - | - | | + | - | + | | B.1.617.2 (Delta) | | - | | A | G | G | G | | B.1.617.2 (Delta) | |
| 138 | - | - | - | | + | - | + | | B.1.617.2 (Delta) | | - | | A | G | G | G | | B.1.617.2 (Delta) | |
| 139 | - | - | - | | + | - | + | | B.1.617.2 (Delta) | | - | | A | G | G | G | | B.1.617.2 (Delta) | |
| 140 | - | - | - | | + | - | + | | B.1.617.2 (Delta) | | - | | A | G | G | G | | B.1.617.2 (Delta) | |
| 141 | - | - | - | | + | - | + | | B.1.617.2 (Delta) | | - | | A | G | G | G | | B.1.617.2 (Delta) | |
| 142 | - | - | - | | + | - | + | | B.1.617.2 (Delta) | | - | | A | G | G | G | | B.1.617.2 (Delta) | |
| 143 | - | - | - | | + | - | + | | B.1.617.2 (Delta) | | - | | A | G | G | G | | B.1.617.2 (Delta) | |
| 144 | - | - | - | | + | - | + | | B.1.617.2 (Delta) | | - | | A | G | G | G | | B.1.617.2 (Delta) | |
| 145 | - | - | - | | + | - | + | | B.1.617.2 (Delta) | | - | | A | G | G | G | | B.1.617.2 (Delta) | |
| 146 | + | - | - | | - | - | - | | B.1.1.7 (Alpha) | | + | | T | T | G | A | | B.1.1.7 (Alpha) | |
| 147 | - | - | - | | + | - | + | | B.1.617.2 (Delta) | | - | | A | G | G | G | | B.1.617.2 (Delta) | |
| 148 | - | - | - | | + | - | + | | B.1.617.2 (Delta) | | - | | A | G | G | G | | B.1.617.2 (Delta) | |
| 149 | - | - | - | | + | - | + | | B.1.617.2 (Delta) | | - | | A | G | G | G | | B.1.617.2 (Delta) | |
| 150 | - | - | - | | + | - | + | | B.1.617.2 (Delta) | | - | | A | G | G | G | | B.1.617.2 (Delta) | |
| 151 | - | - | - | | + | - | + | | B.1.617.2 (Delta) | | - | | A | G | G | G | | B.1.617.2 (Delta) | |
| 152 | - | - | - | | + | - | + | | B.1.617.2 (Delta) | | - | | A | G | G | G | | B.1.617.2 (Delta) | |
| 153 | - | - | - | | + | - | + | | B.1.617.2 (Delta) | | - | | A | G | G | G | | B.1.617.2 (Delta) | |
| 154 | - | - | - | | + | - | + | | B.1.617.2 (Delta) | | - | | A | G | G | G | | B.1.617.2 (Delta) | |
| 155 | - | - | - | | + | - | + | | B.1.617.2 (Delta) | | - | | A | G | G | G | | B.1.617.2 (Delta) | |
| 156 | - | - | - | | + | - | + | | B.1.617.2 (Delta) | | - | | A | G | G | G | | B.1.617.2 (Delta) | |
| 157 | - | - | - | | + | - | + | | B.1.617.2 (Delta) | | - | | A | G | G | G | | B.1.617.2 (Delta) | |
| 158 | - | - | - | | + | - | + | | B.1.617.2 (Delta) | | - | | A | G | G | G | | B.1.617.2 (Delta) | |
| 159 | - | - | - | | + | - | + | | B.1.617.2 (Delta) | | - | | A | G | G | G | | B.1.617.2 (Delta) | |
| 160 | - | - | - | | + | - | + | | B.1.617.2 (Delta) | | - | | A | G | G | G | | B.1.617.2 (Delta) | |
